# Diversity and Use of Edible Grasshoppers, Locusts, Crickets, and Katydids (Orthoptera) in Madagascar

**DOI:** 10.3390/foods8120666

**Published:** 2019-12-10

**Authors:** Joost Van Itterbeeck, Irina N. Rakotomalala Andrianavalona, Faneva I. Rajemison, Johanna F. Rakotondrasoa, Valisoa R. Ralantoarinaivo, Sylvain Hugel, Brian L. Fisher

**Affiliations:** 1 Center for Asian Area Studies, Rikkyo University, 3-34-1 Nishi-Ikebukuro, Toshima, Tokyo 171-8501, Japan; 2 Department of Plant Medicals, Andong National University, 1375 Kyungdong-Street, Andong, Gyeongbuk 36729, Korea; 3 Madagascar Biodiversity Center, Parc Botanique et Zoologique de Tsimbazaza, BP 6257, Antananarivo 101, Madagascar; irinaandrianavalona@gmail.com (I.N.R.A.); iharantsoa.faneva@gmail.com (F.I.R.); 4 Department of Entomology, University of Antananarivo, BP 906, Antananarivo 101, Madagascar; faniryjohanna@gmail.com (J.F.R.); valisoaral96@gmail.com (V.R.R.); 5 Institute of Cellular and Integrative Neuroscience, National Center for Scientific Research (CNRS), University of Strasbourg, 5 rue Blaise Pascal, 67084 Strasbourg, France; hugels@inci-cnrs.unistra.fr; 6 California Academy of Sciences, San Francisco, CA 94118, USA; bfisher@calacademy.org

**Keywords:** insect, entomophagy, biodiversity, food, bioresource, culture

## Abstract

Madagascar has a long history of using Orthoptera as food and feed. Our understanding of the biological diversity of this resource, its contemporary use, and its future potentials in Madagascar is extremely limited. The present study contributes basic knowledge of the biological diversity and local uses of edible Orthoptera in Malagasy food cultures. Data was collected with key informants in 47 localities covering most of the ecoregions of Madagascar and corresponding to 12 of the 19 ethnic groups. Orthoptera are consumed throughout Madagascar. We report 37 edible Orthoptera species, of which 28 are new species records of edible Orthoptera in Madagascar and 24 are new species records of edible Orthoptera in the world. Most species are endemic and occur in farming zones. Children are the primary collectors and consumers of edible Orthoptera. The insects are eaten both as snacks and main meals. Edible Orthoptera are primarily collected casually and marketing is rare, with the notable exceptions of the large cricket *Brachytrupes membranaceus colosseus* and during locust outbreaks (e.g., *Locusta migratoria*). The use of Orthoptera as feed seems rare. Further investigations of cultural and personal preferences are required to assess the future potential roles of Orthoptera in Malagasy food habits.

## 1. Introduction

Madagascar has a long history of using Orthoptera as food and feed. However, our understanding of the biological diversity of this resource, its contemporary use, and its future potentials in Madagascar is extremely limited.

As early as 1617, missionaries and other visitors to Madagascar reported the regular consumption of Orthoptera, especially locusts, by the Malagasy people, and received invitations from the Malagasy people to join them in eating the insects [1]. Some visitors consumed the insects with as good an appetite as the Malagasy [2]. The Malagasy claimed to revenge themselves on these Orthoptera that devastated rice fields and plantations by eating the insects [2,3]. During outbreaks, locusts provided the necessary nutrition for people and livestock (e.g., pigs). Additionally, between harvests, when food supplies were low, Orthoptera and other insects were consumed regularly [1,3]. Locusts were sundried for later use. Malagasy royals consumed the locusts as well. Their most appreciated recipe involved soaking sundried locusts for half an hour in salted water and then frying them in fat. Queen Ranavalona II, who reigned Madagascar in the nineteenth century, had female servants travel to the countryside specifically to collect locusts for her [1,3]. Camboué [3], as well as others [1], considered Orthoptera a potential good source of nutrition for the Malagasy people, and/or their livestock, citing the consumption of Orthoptera in Leviticus in the Bible.

The recent surge of interest in the use of nutritious insects as food and feed may benefit Madagascar. Supporting and advancing the traditional inclusion of insects in Malagasy food cultures, as well as the raising of livestock with insects, may aid the country’s nutritional and economic development, potentially in ecologically sustainable ways [4,5,6]. Edible Orthoptera are among the candidate insects for innovative action. Madagascar’s food production relies heavily on rice cultivation, supplemented with vegetable and fruit crops. Improved agricultural productivity will help to reduce high poverty and food insecurity rates [7]. Rice field ecosystems and other agricultural fields are habitats that globally support high diversities of edible Orthoptera and other edible insects [4,8,9]. One potential strategy is to advance the mechanical control of edible Orthopteran pest insects, which may simultaneously support crop protection and provide a ‘second crop’ in the form of the edible pest insects for food and/or feed [10,11,12]. Another potential strategy is to develop farming technologies to produce local edible Orthoptera for food and/or feed [13,14,15].

The present study contributes to the exploration of edible Orthoptera as candidate insects for edible insect-based nutritional, economic, and ecologically sustainable food production programs. Very few studies have addressed the biological diversity and local uses of edible Orthoptera in contemporary Malagasy food cultures, and these have probably underestimated the diversity of edible Orthopterans [16,17]. Such basic knowledge is of major importance to understand the future potential of edible Orthoptera in Madagascar [18]. Hence, this study aims to (i) identify the edible Orthopteran diversity and explore its geographical distributions and (ii) explore the basic uses of Orthoptera as food and feed in different ethnic groups and across different ecoregions of Madagascar.

## 2. Materials and Methods

### 2.1. Localities and Key Informants

**Figure 1:**
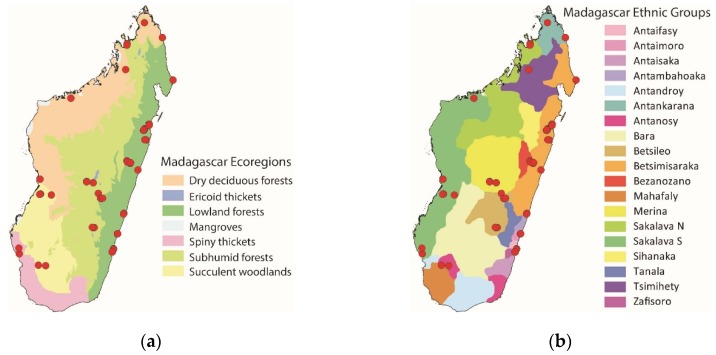
(**a**) Map of Madagascar ecoregions; (**b**) map of Madagascar ethnic groups. Red dots mark the surveyed localities. Particularly in east Madagascar, red dots overlap due to map scale.

Field work was conducted from February to July 2018, in December 2018, and from February to April 2019. We surveyed for edible Orthopteran diversity in 47 localities covering most of the ecoregions of Madagascar and corresponding to 12 of the 19 ethnic groups (Figure 1). All localities were rural villages with rice cultivation as the main subsistence activity. Data was collected with key informants, i.e., rural villagers, who are highly knowledgeable about which insects are edible, where they can be found, how to collect them, and how they are used as food and feed. The key informants were identified via intermediaries, i.e., market vendors, hotel staff, taxi drivers, and agricultural extension offices in rural towns. We explained our research objectives to the intermediaries who then suggested potential key informants and localities and provided contact details and directions. We then met with the potential key informants, thoroughly discussed our research objectives with them, and made a basic assessment of their knowledge by discussing edible insect collection and consumption. Whenever we had doubts about the quality of a potential key informant, we identified another potential key informant (this was rarely necessary). Each locality was then surveyed with the key informants for 1–2 days. All informants gave their consent to participate in the research. Key informants were 28 adults (23 males and 5 females, between about 20 and 60 years old); children sometimes assisted by collecting specimens. Key informants received monetary rewards equivalent to daily wages of manual labor. Research permissions were provided by the Ministry of Environment and Sustainable Development, local authorities (e.g., mayoral offices), and village leaders. A higher number of localities were surveyed in east Madagascar (assessed from February to July 2018) than elsewhere, for two reasons. Firstly, more researchers were available for field work during February–July 2018, which allowed them to split into two teams daily, each accompanied by a key informant, and conduct field work in different sections of a single village and in neighboring villages. Secondly, safety issues and scarcity of transportation limited data collection in central and west Madagascar.

### 2.2. Insect Specimens

Edible Orthoptera specimens were sampled during field trips with key informants in natural areas, agricultural areas, and home gardens. The insects were photographed both in their natural habitat and close up (profile, head, and dorsal). Specimens were sampled by hand, with tweezers, and with sweep nets, and stored in 95% alcohol in vials and Whirl-Pak^®^ bags (Nasco, Fort Atkinson, WI, USA). Specimens are permanently stored in the entomology collections of the California Academy of Sciences, San Francisco, USA. Specimens were identified by Sylvain Hugel, co-author of the present study, with data from Dirsh and Descamps [19]. Geographical distributions and threats to crops were determine by referring to Braud et al. [20]. Calculations were performed with Microsoft Excel.

### 2.3. Edible Orthoptera Use

Key informants were interviewed on edible Orthoptera use during the edible insect specimen collection field trips. We recorded: (i) vernacular names, (ii) collection methods, (iii) gender and age of collectors and consumers, (iv) consumption mode (i.e., snack or main meal), (v) food preservation methods, (vi) frequencies and quantities of collection and consumption, (vii) marketing, (viii) animal feed uses, (ix) medicinal uses, and (x) taboos. We emphasize that we report a qualitative overview only. Preferences in the use of edible Orthoptera vary among ethnic groups and individuals within ethnic groups. Due to the use of a limited number of informants, it is not in the scope of the present study to make inferences of cultural and personal preferences.

## 3. Results

### 3.1. Edible Orthopteran Diversity

**Figure 2:**
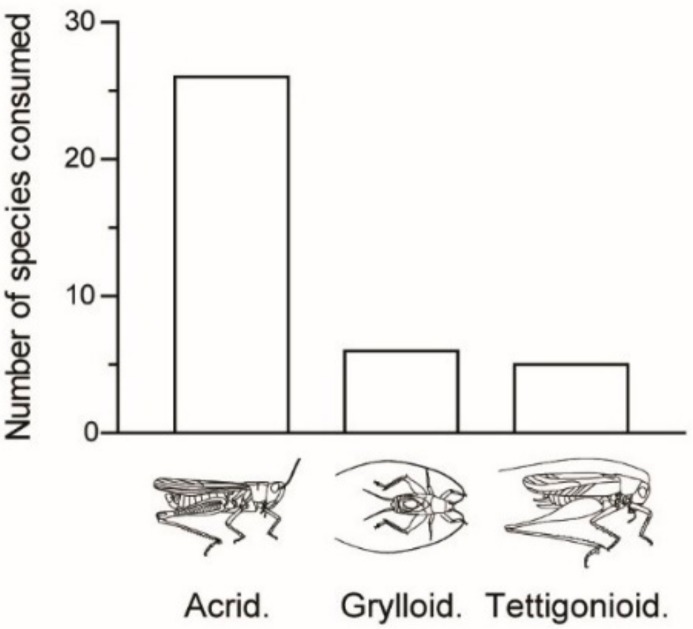
Number of edible Orthoptera species as a function of systematics. Legend: Acrid., Acrididae; Grylloid., Grylloidea; Tettigonioid., Tettigonioidea.

We recorded 37 species of edible Orthoptera, of which 31 were able to be identified to species level and six to either genus or subfamily level (Table 1). This edible Orthopteran diversity corresponds to 3% of the recorded Orthopteran diversity in Madagascar (ca. 700 species, likely underestimated). These 37 species are comprised of 26 species of grasshoppers (Caelifera) and 11 species of crickets and katydids (Ensifera; Figure 2). Most of the edible Caelifera belong to the Acrididae (23 out of 26 species). The edible Ensifera includes Tettigonioidea (bush crickets/katydids, six species) and Grylloidea (crickets, five species).

**Figure 3:**
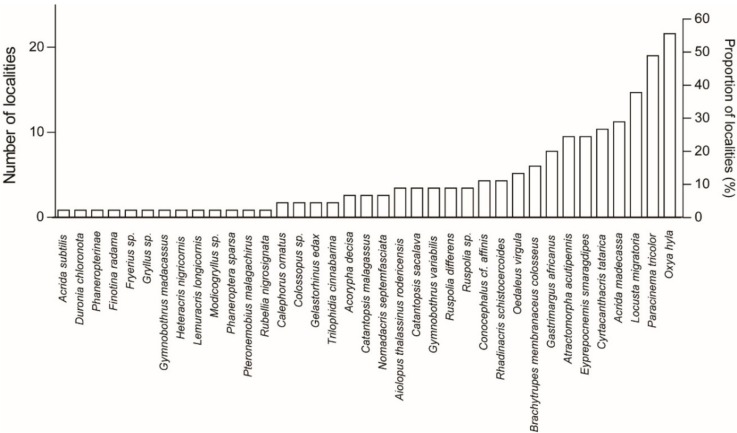
Number of localities per edible Orthoptera species.

The five most recorded species were found to be *Oxya hyla* (25 localities in three ecoregions: lowland forests, subhumid forests, and succulent woodlands; nine ethnic groups), *Paracinema tricolor* (22 localities in three ecoregions: lowland forests, subhumid forests, and succulent woodlands; nine ethnic groups), *Locusta migratoria* (17 localities in five ecoregions: lowland forests, subhumid forests, succulent woodlands, dry deciduous forests, and spiny thickets; eight ethnic groups), *Acrida madecassa* (13 localities in five ecoregions: lowland forests, subhumid forests, succulent woodlands, dry deciduous forests, and spiny thickets; six ethnic groups), and *Cyrtacanthacris tatarica* (12 localities in five ecoregions: lowland forests, subhumid forests, succulent woodlands, dry deciduous forests, and spiny thickets; seven ethnic groups). Of the 37 recorded species, 14 species were sampled only once (i.e., one locality per species; Figure 3). An annotated list of edible Orthoptera is provided in Section 3.3.

The total count of 37 species is conservative. In cases of unidentified species, all specimens belonging to a single genus or subfamily were counted as a single species. We thus have not counted *Acrida* sp. and *Brachytrupes* sp. as a different species from *Acrida subtilis* and *A. madecassa*, and from *Brachytrupes membranaceus colosseus*, respectively, since only juveniles were collected and could not be identified to species level. *Ruspolia* sp., however, was considered a separate species since some adults were collected and the specimens were definitely not *Ruspolia differens*. Both juveniles and adults are edible. Among the 279 species-sorted batches examined for the present work, 87% contained at least some juveniles. Juveniles are smaller than adults but cannot escape by flying.

**Figure 4:**
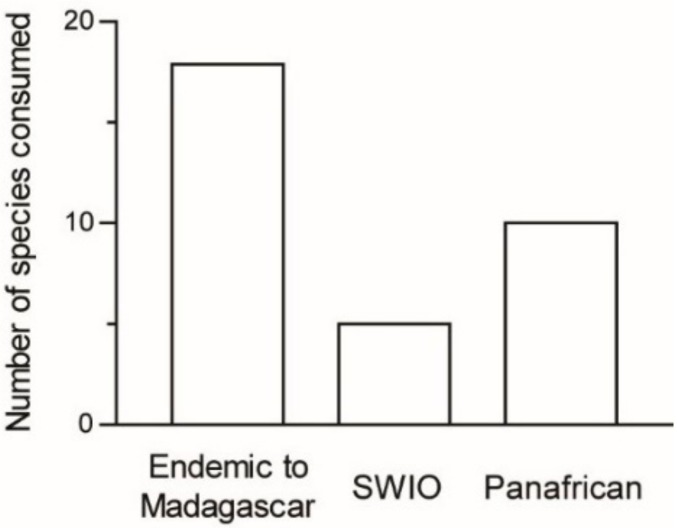
Number of edible Orthoptera species as a function of their distribution in Madagascar, South Western Indian Ocean (SWIO), and African areas.

Most of the recorded species are endemic to the Malagasy area: 18 species (56%) are endemic to Madagascar and five species (16%) occur also in nearby islands, whereas nine species (28%) are widely distributed across Africa (*n* = 32 species; Figure 4).

Somewhat surprisingly, two Caelifera species, *Rubellia nigrosignata* and *Atractomorpha acutipennis*, belong to the Pyrgomorphidae, a family with many species considered to be unpleasant-tasting or -smelling, or even toxic.

**Figure 5:**
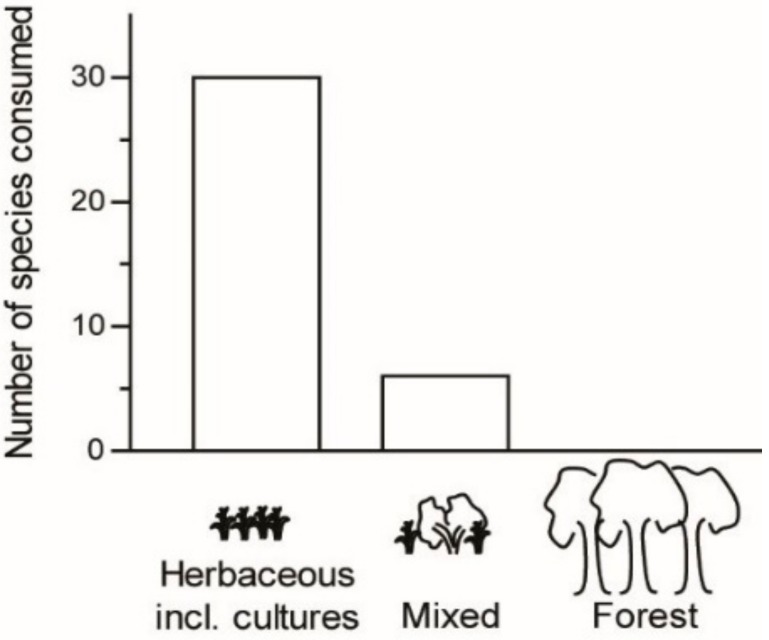
Number of edible Orthoptera species as a function of their habitat.

**Figure 6:**
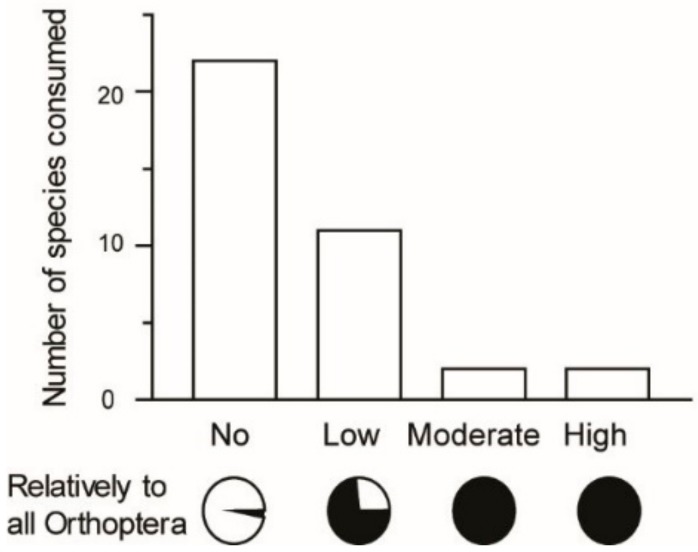
Number of edible Orthoptera species as a function of their threat to crops.

Most edible Orthoptera were found to occur in habitats with herbaceous plant cover, including farming zones (83%); few were found to occur in areas mixed with taller plant cover (17%); and none were found to occur in forest habitat (*n* = 36; Figure 5). The majority of Orthoptera species recorded as edible were no threat to crops (22 species, 59% of recorded edible Orthoptera in the present study; Figure 6). Fifteen species were found to be a threat to crops, of which two species posed a high threat (5%), two species a moderate threat (5%), and 11 species a low threat (30%; *n* = 37 species). Our record indicates that threat level is associated with use as food: all species representing a high threat (100%) and a moderate threat (100%) to crops were reported as edible, whereas 73% of species posing a low threat were reported as edible and 3% of species posing no threat were reported as edible (*n* = 37 species). Rice field ecosystems, other agricultural fields, and fallow land near agricultural fields are the main sources of the edible Orthoptera bioresource used in Madagascar.

### 3.2. Edible Orthoptera Uses

The highest edible Orthopteran diversity was recorded among the Merina in the subhumid forests ecoregion (22 species, 59% of recorded edible Orthoptera in the present study), the Betsileo in the subhumid forests ecoregion (20 species, 54%), and the Betsimsaraka in the lowland forest ecoregion (18 species, 49%). The lowest edible Orthopteran diversity was recorded among the Sakalava N (North) and Tsimihety, both in the dry deciduous forests ecoregion (two species each, 5%), the Bezanozano in the lowland forests ecoregion (three species, 8%), the Antankarana in the dry deciduous forests ecoregion (four species, 11%), and the Antanosy in the succulent woodlands ecoregion (four species, 11%). The most widespread used edible Orthoptera were found to be *P. tricolor* and *O. hyla* (nine ethnic groups each), *L. migratoria* (eight ethnic groups), and *C. tatarica* and *Eyprepocnemis smaragdipes* (seven ethnic groups each). The most common vernacular name observed for edible Orthoptera was *valala*, with some other names also mentioned (Table 1).

Edible Orthoptera are present throughout the year with roughly two peak abundance periods from October–January and March–June. Edible Orthoptera are primarily collected casually in Madagascar. All ethnicities collect the insects only by hand. Children are the primary collectors and consumers of Orthoptera in all ethnicities, although adults may also collect and eat them. No gender differences seem to be present in the collection and consumption of Orthoptera. Collecting edible Orthoptera is a common game for children playing in the rice fields or walking home from school. The children then prepare and eat the Orthoptera themselves or have their mothers prepare the insects for them (mothers may then snack on the Orthoptera with their children). Adults mostly collect Orthoptera during their peak availability but may also collect them outside of the peak availability periods, notably while working in the rice fields. When adults collect edible Orthoptera, they may prefer to give them to their children for playing and for eating. Orthoptera are eaten as a snack and as part of a main meal. The wings, legs, head, and intestines may be removed before the insects are prepared by grilling, frying, and frying after being cooked in water. The insects may be seasoned with salt. Some recipes to prepare Orthoptera in Madagascar are provided in the Supplementary File. We do not have clear information about collection and consumption frequencies and quantities. Orthoptera may be used as animal feed (e.g., poultry) but this seems rare. Preservation of Orthoptera is very rare. Only locusts in south Madagascar are dried when outbreaks occur.

Marketing is rare due to low numbers of Orthoptera that are collected casually and due to time constraints to increase Orthoptera yields. Some exceptions occur. Locusts are marketed during outbreaks in south and central Madagascar, and adults may then collect locusts even daily and opportunistically to eat and market them. The large-sized *B. membranaceus colosseus* may be intentionally collected for marketing purposes in west Madagascar. This cricket may also be specifically sought after when meat is expensive and when no other rice accompaniments are available. The cricket is sold for 100–200 Malagasy Ariary (0.027–0.055 US$) per individual body and for 500–1500 Malagasy Ariary (0.14–0.41 US$) per *kapoaka*, a tin can of about 400 mL commonly used as a unit of measurement for selling produce. The level of edible Orthoptera marketing seems to be lower than in some other countries with Orthoptera consumption, such as Togo [21], Laos [22], and Japan [23,24].

Malagasy Muslims, who live primarily in north-west Madagascar, do not eat any insects for religious reasons. The eating of *sakoririka*, a vernacular name for Orthoptera that at least includes the genus *Ruspolia*, is taboo among the Antaisaka ethnic group: the insect is only consumed as a medicine by infants who refrain from speech, or are incapable of speech, to help them to speak. This taboo may not be shared by all ethnicities. The taboo was mentioned by members of the Antaisaka ethnic group of southeast Madagascar who had migrated to the regions commonly associated with the Sakalava ethnic groups in west Madagascar. No other medicinal use of Orthoptera was mentioned in any surveyed localities. Mole crickets are not considered edible due to a widespread cultural belief. They are called “zazavery”, which means “lost child”. The Malagasy people regularly encounter mole crickets in wet soils, e.g., along riverbanks while cultivating vegetables or cutting wood. However, these mole crickets quickly disappear as they dig themselves into the wet soil to hide. The Malagasy liken this act of disappearing to the behavior of children who may suddenly wander off to play without notifying their parents. The large Pyrgomorphidae grasshopper *Phymateus saxosus* is renowned as inedible in Madagascar, as are some other Pyrgomorphidae. It has aposematic coloration and produces an unpleasant smell when disturbed. This grasshopper is named “dog’s grasshopper” (*valalan’alika* or *valalan’amboa*) and Malagasy people say that “even dogs don’t eat them”.

### 3.3. Annotated List of Edible Orthoptera

#### 3.3.1. *Caelifera*, *Acridinae*

**Figure 7:**
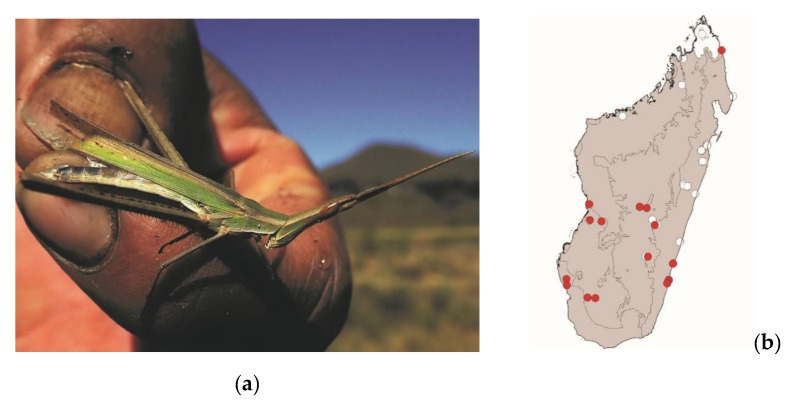
(**a**) *Acrida madecassa* (picture courtesy of Faneva I. Rajemison); (**b**) distribution map—red dots mark the localities where *A. madecassa* specimens were sampled in the present study and the grey background indicates the ecoregions where *A. madecassa* occurs.

*Acrida madecassa* (Brancsik, 1892) (Figure 7) and *Acrida subtilis* (Burr, 1902)

These elongated grasshoppers measure 35–75 mm and are endemic to Madagascar. They are widespread in most of Madagascar but are absent from the northern tip of the island. They live in tall grassy areas, including within or near crops. These species are active by daylight and pose no threat to crops. They are consumed in the southern half of Madagascar from the coasts to the central plateau (Figure 3). Despite their strong sexual dimorphism (males measuring 35–45 mm and females 60–75 mm), both males and females are consumed.

*Aiolopus thalassinus rodericensis* (Butler, 1876)

This grasshopper measures 15–25 mm. It is present in virtually every non-forested area of Madagascar except the east coast and surrounding islands. *Aiolopus thalassinus rodericensis* live in bare soil and grassy areas. This species is active by daylight and poses no threat to crops. It is consumed in the central part of Madagascar (from the west coast to the central plateau).

*Duronia chloronota* (Stål, 1876)

This grasshopper measures 20–45 mm. It is present in non-forested areas of Madagascar, except along the east coast. It lives in grassy areas. This species is active by daylight and poses no threat to crops. The consumption of this species was recorded for one single locality in west Madagascar.

*Calephorus ornatus* (Walker, 1870)

This small grasshopper measures 10–25 mm. It is widespread in wet grassy areas of Madagascar, particularly near rivers and lakes and in wetlands, including rice fields. This species is active by daylight and poses no threat to crops. It is consumed in the southeast of Madagascar.

*Gymnobothrus madacassus* (Bruner, 1910) and Gymnobothrus variabilis (Bruner, 1910)

*Gymnobothrus* species measure 12–25 mm. The genus *Gymnobothrus* is found widely across Madagascar and the Comoros. They live in bare soil and grassy areas. These species are active by daylight and pose no threat to crops. These species are consumed in central and east Madagascar.

#### 3.3.2. *Caelifera*, *Calliptaminae*

*Acorypha decisa* (Walker, 1870)

This grasshopper measures 20–35 mm. It is endemic to Madagascar and widespread across most of the island save the northern tip. It lives in grassy areas. The species is active by daylight and poses no threat to crops. This species is consumed in central Madagascar between Antananarivo and Fianarantsoa.

#### 3.3.3. *Caelifera*, *Catantopinae*

*Catantopsis malagassus* (Karny, 1907) and *Catantopsis sacalava* (Brancsik, 1892)

*Catantopsis* species measure 15–30 mm. The genus *Catantopsis* is endemic to Madagascar and the Comoros, where it is widely present. These grasshoppers live in grassy areas as well as shrublands. These species are active by daylight and pose no threat to crops. Localities where *Catantopsis* are consumed are scattered across Madagascar.

#### 3.3.4. *Caelifera*, *Cyrtacanthacridinae*

*Cyrtacanthacris tatarica* (Linnaeus, 1758)

**Figure 8:**
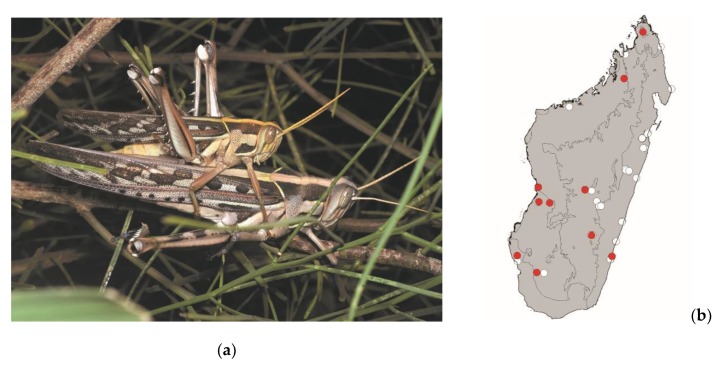
(**a**) *Cyrtacanthacris tatarica* (picture courtesy of Sylvain Hugel); (**b**) distribution map—red dots mark the localities where *C. tatarica* specimens were sampled in the present study, and the grey background indicates the ecoregions where *C. tatarica* occurs.

This large locust measures 40–65 mm. It is widespread in Madagascar, the Comoros, and continental Africa. It lives in areas with tall grass as well as shrublands. This species is active by daylight. It represents a low threat to crops. Localities where *C. tatarica* is consumed are scattered across Madagascar, in both lowlands and highlands (Figure 8).

*Rhadinacris schistocercoides* (Brancsik, 1892)

This large locust measures 30–50 mm. It is endemic to Madagascar. It is widespread across the island, except along the east coast. It lives in areas with tall grass as well as shrublands. This species is active by daylight and poses no threat to crops. This species is consumed in central Madagascar, from the east to the central plateau.

*Finotina radama* (Brancsik, 1892)

This large locust measures 38–60 mm. It is endemic to Madagascar. It is widespread across the island except along the east coast. It lives in areas with tall grass as well as shrublands. This species is active by daylight and poses no threat to crops. The consumption of this species was recorded for one locality in central Madagascar.

*Nomadacris septemfasciata* (Serville, 1838)

This large plague locust measures 60–75 mm. It is widespread in continental Africa, Madagascar, and islands around Madagascar. It lives in areas with tall grass as well as shrublands. This species is active by daylight and poses a high threat to crops. Localities where *N. septemfasciata* is consumed are scattered across Madagascar.

#### 3.3.5. *Caelifera*, *Eyprepocnemidinae*

*Heteracris nigricornis* (Saussure, 1899)

This grasshopper measures 28–50 mm. It is endemic to Madagascar. It is widespread across the island except in the north and southwest. It lives in areas with tall grass as well as shrublands. This species is active by daylight and poses no threat to crops. The consumption of this species was recorded for one locality in central Madagascar.

*Eyprepocnemis smaragdipes* (Bruner, 1910)

**Figure 9:**
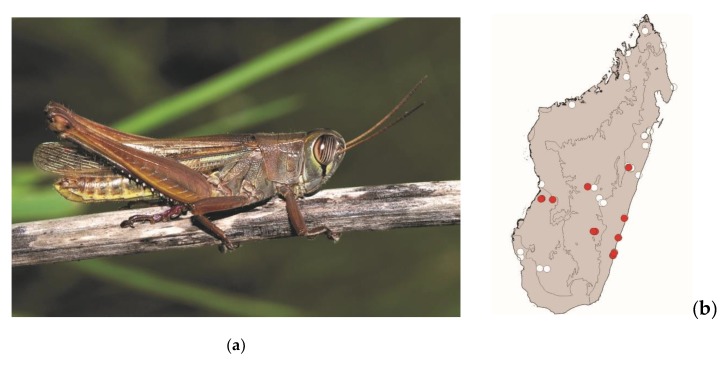
(**a**) *Eyprepocnemis smaragdipes* (picture courtesy of Sylvain Hugel); (**b**) distribution map—red dots mark the localities where the current study sampled *E. smaragdipes* specimens, and the grey background indicates the ecoregions where *E. smaragdipes* occurs.

This grasshopper measures 20–40 mm. It is widespread in continental Africa, Madagascar, and islands around Madagascar. It lives in areas with tall grass. This species is active by daylight and poses a low threat to crops. This species is consumed in central Madagascar, from the east to the west coast (Figure 9).

#### 3.3.6. *Caelifera*, *Gomphocerinae*

*Gelastorhinus edax* (Saussure, 1888)

This grasshopper measures 22–45 mm. It is endemic and widespread in Madagascar. It lives in grassy areas. This species is active by daylight and poses no threat to crops. This species is consumed in central Madagascar, between Antananarivo and Fianarantsoa.

#### 3.3.7. *Caelifera*, *Oedipodinae*

*Paracinema tricolor* (Thunberg, 1815)

**Figure 10:**
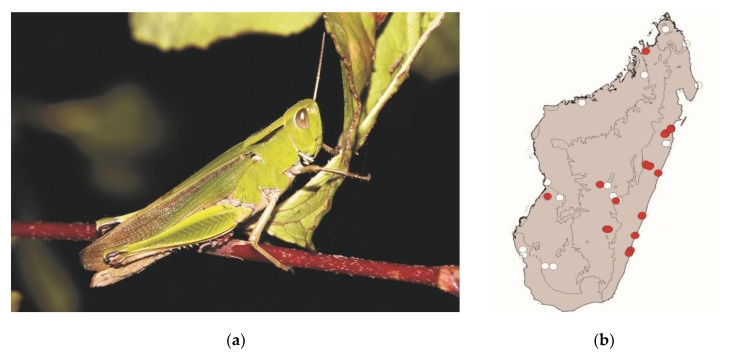
(**a**) *Paracinema tricolor* (picture courtesy of Sylvain Hugel); (**b**) distribution map—red dots mark the localities where the current study sampled *P. tricolor* specimens, and the grey background indicates the ecoregions where *P. tricolor* occurs.

This grasshopper measures 15–40 mm. It is widespread in continental Africa and Madagascar. It is among the most common grasshoppers found in wet, grassy areas of Madagascar, particularly near rivers and lakes and in wetlands, including rice fields. This species is active by daylight and poses a moderate threat to crops. This species is among the most widely consumed in Madagascar (Figure 10).

*Gastrimargus africanus* (Saussure, 1888)

**Figure 11:**
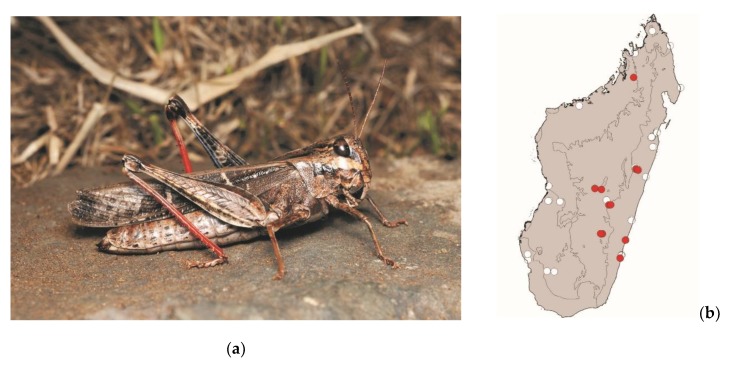
(**a**) *Gastrimargus africanus* (picture courtesy of Sylvain Hugel); (**b**) distribution map—red dots mark the localities where the current study sampled *G. africanus* specimens, and the grey background indicates the ecoregions where *G. africanus* occurs.

This relatively large locust measures 22–40 mm. It is widespread in continental Africa, Madagascar, and the southwestern Indian Ocean islands. In Madagascar it is widespread in grassy areas, including savannas maintained by fires. This species is active by daylight and poses a moderate threat to crops. Localities where *G. africanus* is consumed are mostly in the central and eastern parts of Madagascar (Figure 11).

*Lemuracris longicornis* (Dirsh, 1966)

This grasshopper measures > 30 mm. It is endemic to the east of Madagascar. It lives in areas with tall grass as well as shrublands. This species is active by daylight and poses no threat to crops. The consumption of this species was recorded for one locality in east Madagascar.

*Locusta migratoria* (Linnaeus, 1758)

**Figure 12:**
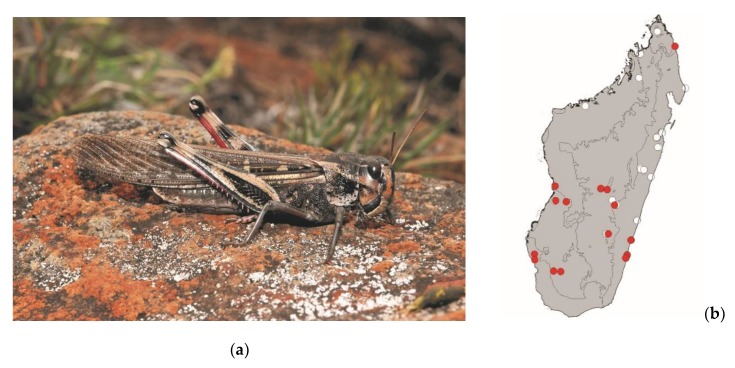
(**a**) *Locusta migratoria* (picture courtesy of Sylvain Hugel); (**b**) distribution map—red dots mark the localities where the current study sampled *L. migratoria* specimens, and the grey background indicates the ecoregions where *L. migratoria* occurs.

This large plague locust measures 35–55 mm. It is widespread in continental Africa, Madagascar, and the southwestern Indian Ocean islands. It occurs in grassy areas of Madagascar and is frequently found in and around crops. This species is active by daylight and poses a high threat to crops. *Locusta migratoria* is mostly consumed in the southern half of Madagascar (Figure 12).

*Oedaleus virgula* (Snellen van Vollenhoven, 1869)

This grasshopper measures 13–32 mm. It is widespread in continental Africa and Madagascar. It occurs in bare soil and grassy areas of Madagascar. It is frequently found in and around crops. This species is active by daylight and poses no threat to crops. *Oedaleus virgula* is mostly consumed in the eastern half of Madagascar, from the central plateau to the east coast.

*Trilophidia cinnabarina* (Brancsik, 1892)

This small grasshopper measures 14–25 mm. It is endemic and widespread in Madagascar. It occurs in bare soil and grassy areas of Madagascar and is frequently found in and around crops. This species is active by daylight and poses no threat to crops. Consumption of *T. cinnabarina* was recorded for two localities in central Madagascar.

#### 3.3.8. *Caelifera*, *Oxyinae*

*Oxya hyla* (Serville, 1831)

**Figure 13:**
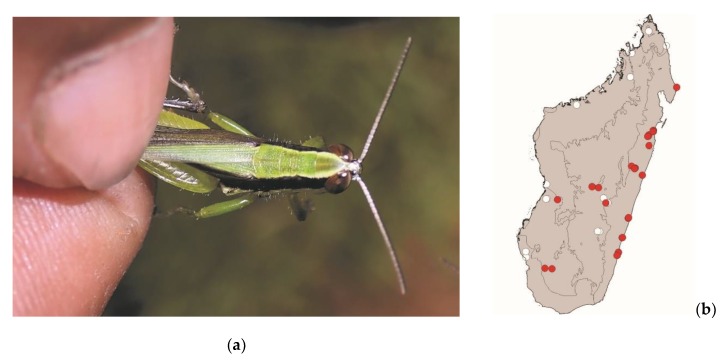
(**a**) *Oxya hyla* (picture courtesy of Sylvain Hugel); (**b**) distribution map—red dots mark the localities where the current study sampled *O. hyla* specimens, and the grey background indicates the ecoregions where *O. hyla* occurs.

This grasshopper measures 20–31 mm. It is widespread in continental Africa and Madagascar. It is among the most common grasshoppers in wet, grassy areas of Madagascar, particularly near rivers and lakes and in wetlands, including rice fields. This species is active by daylight. It represents a low-to-moderate threat to crops. This species is consumed in the central plateau and along the east coast. This species is among the most widely consumed in Madagascar (Figure 13).

#### 3.3.9. *Caelifera*, *Pyrgomorphinae*

Members of Pyrgomorphidae feed on toxic plants such as Apocynaceae and sequester secondary metabolites which have significant effects on the physiology of vertebrates via cardenolide steroids and pyrrolizidine alkaloids [25,26,27,28,29]. These Pyrgomorphidae often display aposematic coloration and some possess specialized glands or devices allowing the release of these metabolites upon disturbance.

*Atractomorpha acutipennis* (Guérin-Méneville, 1844)

**Figure 14:**
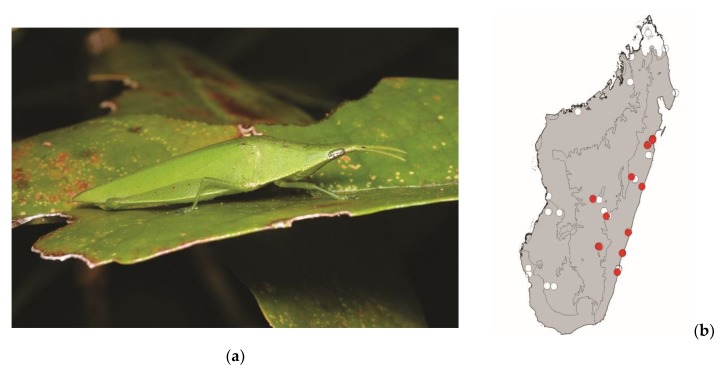
(**a**) *Atractomorpha acutipennis* (picture courtesy of Sylvain Hugel); (**b**) distribution map—red dots mark the localities where the current study sampled *A. acutipennis* specimens, and the grey background indicates the ecoregions where *A. acutipennis* occurs.

This grasshopper measures 15-40 mm. It is widespread in continental Africa, Madagascar, and the Comoros. This grasshopper lives in areas with tall grass as well as shrublands. The species is active by daylight and poses no threat to crops. This species is very widely consumed in the central plateau and along the east coast (Figure 14). *Atractomorpha acutipennis* does not have an aposematic coloration, emits an odor less strong than that of most Pyrgomorphidae, and does not feed primarily on toxic plants.

*Rubellia nigrosignata* (Stål, 1875)

This grasshopper measures 15–30 mm. It is endemic to Madagascar. This grasshopper lives in areas with tall grass as well as shrublands. The species is active by daylight and poses a low threat to crops. Consumption of *R. nigrosignata* was recorded for one locality in east Madagascar. The insect produces an unpleasant smell when manipulated. *Rubellia nigrosignata* feeds on a variety of plants, though not primarily toxic ones.

#### 3.3.10. *Ensifera*, *Grylloidea*

*Gryllus* sp.

The consumption of a juvenile of one large ground cricket (20 mm) was recorded for one locality on the east coast of Madagascar. These crickets are nocturnal and represent no threat to crops. Juveniles of *Gryllus* sp. Cannot be identified at the species level.

*Modicogryllus* sp.

The consumption of a medium-sized female ground cricket (10 mm) was recorded for one locality on the east coast of Madagascar. These crickets are nocturnal and represent no threat to crops. Females of *Modicogryllus* sp. Cannot be identified at the species level.

*Brachytrupes membranaceus colosseus* (Saussure, 1899)

**Figure 15:**
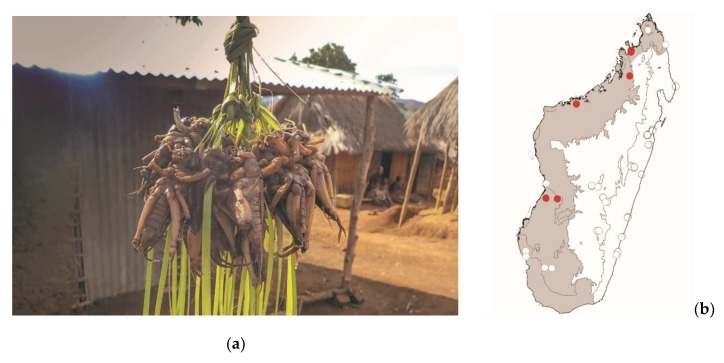
(**a**) *Brachytrupes membranaceus colosseus* (picture courtesy of Faneva I. Rajemison); (**b**) distribution map—red dots mark the localities where the current study sampled *B. membranaceus colosseus* specimens, and the grey background indicates the ecoregions where *B. membranaceus colosseus* occurs.

These large ground crickets are widespread in sandy areas of the west of Madagascar. Several endemic species of *Brachytrupes* sp. Occur in Madagascar and are in need of taxonomic review. These large crickets may represent a low threat to crops. *Brachytrupes* sp. Dig burrows at the entrance of which males produce a loud call at dusk. These crickets are consumed in various localities in the west of Madagascar (Figure 15).

*Fryerius* sp.

The consumption of a large female tree cricket (>20 mm) was recorded for one locality. These crickets are nocturnal and represent no threat to crops. Females of *Fryerius* sp. cannot be identified at the species level.

*Pteronemobius* malagachus (Saussure, 1877)

The consumption of a small ground cricket (>10 mm) was recorded for one locality on the east coast of Madagascar. *Pteronemobius malagachus* is frequently found in relatively wet grassy areas of Madagascar and its surrounding islands, including in and around rice fields. This cricket is both diurnal and nocturnal and represents no threat to crops.

#### 3.3.11. *Ensifera*, *Tettigoniidae*

*Conocephalus affinis* (Redtenbacher, 1891)

This katydid measures 20–30 mm. It occurs in areas with tall grass all over Madagascar, including cultivated lands. This katydid is both diurnal and nocturnal and poses no threat to crops. *Conocephalus affinis* is consumed in various localities in the east of Madagascar.

*Ruspolia differens* and other *Ruspolia* sp.

*Ruspolia* sp. measure 30–40 mm. They occur in areas with tall grass all over Madagascar, including cultivated lands. These katydids are mostly nocturnal and may represent a low threat to crops (particularly *R. differens*). *Ruspolia* sp. are consumed in various localities from the central plateau to the east of Madagascar.

*Colossopus* sp.

*Colossopus* sp. are large nocturnal katydids living in areas with shrubs and trees. Adults can reach up to 60 mm. Some species occur in and around cultivated areas. Consumption of *Colossopus* sp. has been recorded in various localities from the central plateau. The collected samples were juveniles and not identifiable at the species level.

*Phaneroptera sparsa* (Stål, 1857) and other Phaneropterinae

*Phaneroptera sparsa* is a small katydid. It is widespread in tropical Africa and across Madagascar and the Comoros. It is mostly nocturnal and lives in areas with tall grass and/or shrubs, including cultivated lands. Consumption of *Phaneroptera sparsa* and another non-adult Phaneropterinae were recorded for two localities in the central plateau.

*Odontolakis* sp.

We have not recorded the consumption of *Odontolakis* species in the present work, but *Odontolakis sexpunctata* bush crickets have previously been recorded as edible by Randrianandrasana and Berenbaum [17].

## 4. Discussion

In this work, we contribute 28 new species records of edible Orthoptera in Madagascar. Of our 31 edible Orthoptera identified to species level, only three species have been reported as edible in the literature on Madagascar: *L. migratoria*, *Nomadacris septemfasciata*, and *B. membranaceus* [1,16,17]. Of all reported edible Orthoptera in Madagascar, only *Odontolakis sexpunctata*, reported by Randrianandrasana and Berenbaum [17], is not recorded in the present study. Additionally, of those 28 new species records, 24 are new species records of edible Orthoptera in the world and have not been reported as edible in the world list of edible insects by Jongema [30].

There are 14 species that we only sampled in one locality per species (Figure 2). This may be due to a generally low abundance of the species in an area and/or due to a low abundance of the species at the time of sampling (e.g., sampling may have occurred outside the peak of seasonal abundance).

The order Orthoptera is divided into two sub-orders: Caelifera (including grasshoppers) and Ensifera (including katydids and crickets). Most edible Orthoptera of Madagascar belong to Caelifera Acridoidea (grasshoppers). These represent 70% of the edible species, with the other 30% corresponding to Ensifera. This may be linked to the predominant timing of the activity of each group. All edible Caelifera recorded in the present study are active by daylight and are therefore easier to spot, while most Ensifera are concealed at that time. Although we focused on sampling insects during daylight hours, a methodological bias is unlikely. Our key informants indicate that edible Orthoptera are mostly collected while playing in the fields and while walking home from school in the case of children, and when outbreaks occur and when working in the fields in the case of adults. These activities occur during daylight hours. In addition, the large cricket *B. membranaceus colosseus* is collected during the day by locating entrance holes and digging it out of the ground. During the last hours of the day, entrance holes are sometimes spotted by listening for calling males. Edible insect collection after sundown does not seem to occur.

Rice field and grassland ecosystems are abundant in Madagascar and provide contemporary Malagasy rural people with the bioresource of edible Orthoptera, as they have throughout Malagasy history. Little may have changed about Orthoptera consumption habits in comparison to a century ago [1,2,3]. Edible Orthoptera have a place in Malagasy cultures throughout the entire country. What remains to be explored is the significance of edible Orthoptera in contemporary Malagasy food cultures and whether edible Orthoptera can continue to play a role in Malagasy food cultures in the future. Key aspects that require investigation include: (i) the determinants of casual collecting as the main contemporary strategy to collect edible Orthoptera (e.g., ecological influences, cultural influences, and personal influences); (ii) contemporary dietary contributions of edible Orthoptera in terms of consumption frequencies, consumption quantities, and nutrients, in comparison to other foods; (iii) contemporary cultural preferences and personal preferences with regard to Orthopteran species as food and with regard to the place of edible Orthoptera within dietary habits (e.g., snack or main meal); (iv) desirability for a more enterprising edible Orthoptera collection strategy for human food and/or animal feed (e.g., increased time allocation, improved harvesting techniques, and adoption of farming techniques); and (v) desirability for a prominent role of edible Orthoptera in local food cultures (e.g., increased consumption frequency and increased consumption quantity). These key aspects need to be investigated in all 19 different ethnicities and the ecoregions they inhabit. We used a small number of key informants per locality, ethnicity, and ecoregion, and therefore, generalizations for ethnicities cannot and should not be made based on our findings. We emphasize the qualitative character of the information provided.

The present study contributes to future research and development programs investigating the use of insects as human food and animal feed by increasing our knowledge of edible Orthoptera diversity, the geographical distribution of edible Orthoptera species, and the basic uses of edible Orthoptera species, by different ethnic groups and in different ecoregions in Madagascar.

## Figures and Tables

**Table 1:** Overview of the 37 edible Orthoptera species in Madagascar recorded in the present study.

Genus and Species	Threat Level to Crops	Ethnic Groups													Vernacular Names
		Antaifasy	Antaimoro	Antankarana	Antanosy	Betsileo	Betsimisaraka	Bezanozano	Merina	Sakalava N	Sakalava S	Tsimihety	Zafisoro	Total	
**CAELIFERA** (grasshoppers, locusts)															
*Locusta migratoria*	+++	X	X	X	X	X			X		X		X	8	Valala, Kijeja, Mendry
*Nomadacris septemfasciata*	+++			X					X					2	Valala, Sipanga
***Gastrimargus africanus***	++		X			X	X		X			X	X	6	Valala
***Paracinema tricolor***	++	X	X			X	X	X	X	X	X		X	9	Valala
***Acorypha decisa***	+					X			X					2	Valala
***Acrida madecassa***	+				X	X	X		X		X		X	6	Valala, Kesoly
***Catantopsis malagassus***	+					X	X		X					3	Valala
***Catantopsis sacalava***	+								X	X	X			3	Valala
***Cyrtacanthacris tatarica***	+	X		X	X	X			X		X	X		7	N/A
***Eyprepocnemis smaragdipes***	+	X	X			X	X		X		X		X	7	Valala
***Finotina radama***	+								X					1	Valala
***Oedaleus virgula***	+	X		X		X	X		X		X			6	Valala
***Oxya hyla***	+	X	X		X	X	X	X	X		X		X	9	Valala
***Rhadinacris schistocercoides***	+								X		X			2	Valala, Sipanga
***Rubellia nigrosignata***	+						X							1	N/A
*Acrida* sp.						X								1	Valala
***Acrida subtilis***						X								1	Valala
***Aiolopus thalassinus rodericensis***									X		X			2	Valala
***Atractomorpha acutipennis***			X			X	X	X	X				X	6	Valala, Sompatra
***Calephorus ornatus***		X				X			X					3	Valala
***Duronia chloronota***											X			1	Kelimavo
***Gelastorhinus edax***						X			X					2	Valala
***Gymnobothrus madacassus***							X							1	Valala
***Gymnobothrus variabilis***		X	X			X								3	Valala
***Heteracris nigricornis***									X					1	Valala
***Lemuracris longicornis***							X							1	N/A
***Trilophidia cinnabarina***						X			X					2	Valala
**ENSIFERA** (crickets, katydids)															
*Brachytrupes* sp.										X		X		2	Sahobaka
*Brachytrupes membranaceus colosseus*											X			1	Sahobaka
*Colossopus* sp.						X			X					2	Sakova
***Conocephalus*** ** cf. *affinis***			X				X							2	Valala
*Phaneropterinae* sp.							X							1	Kisovasova
*Fryerius* sp.							X							1	N/A
*Gryllus* sp.							X							1	N/A
*Modicogryllus* sp.						X								1	N/A
***Phaneroptera sparsa***						X								1	Kisovasova
***Pteronemobius malagachirus***							X							1	N/A
***Ruspolia differens***							X		X				X	3	Valala, Sakoririka
*Ruspolia* sp.			X			X	X				X			4	Valala, Sakoririka
**Total 37**		8	9	4	4	20	18	3	22	2	13	2	8		

Bold = new species records of edible Orthoptera in Madagascar; bold and underlined = new species records of edible Orthoptera in the world (see references in text). Threat level to crops: +++ = high, ++ = moderate, + = low, and blank = no threat. *Acrida* sp. and *Brachytrupes* sp. have been excluded from the total counts of edible Orthoptera species since we have not counted them as different species from *Acrida* subtilis and *A. madecassa*, and from *Brachytrupes membranaceus colosseus*, respectively (see text).

## Supplementary Materials

The following are available online at https://www.mdpi.com/2304-8158/8/12/666/s1. The Supplementary File provides recipes used in Madagascar to prepare Orthoptera.
