# Supplementary material for: Diversity and Use of Edible Grasshoppers, Locusts, Crickets, and Katydids (Orthoptera) in Madagascar
Source: Foods. 2019 Dec 10;8(12):666. doi: 10.3390/foods8120666 (PMC6963331; doi:10.3390/foods8120666)
Supplement: Supplementary file 1 [file foods-08-00666-s001.pdf]

Supplementary File: Recipes used to prepare Orthoptera in Madagascar.

Orthoptera are eaten either as snacks or part of a main meal. We list below some of the recipes for preparing these insects that we encountered during our surveys.

Preparation of Orthoptera is simple, as it depends on the ingredients available in villages. The wings, legs, head, and intestines may be removed before the insects are prepared by grilling, frying, and frying after being cooked in water. Preparation differences occur between locations and appear to depend on the species. Table 1 below illustrates this variation with some examples.

Table 1: Example of recipes used at different locations

**Location**	**Species**	**Local name**	**Collected/Sold**	**Purpose**	**Dish**
Manakara	*Conocephalus affinis*	Apanga tsiriry	Collected	Snack<br>Lunch	1
Vavatenina	*Paracinema tricolor*	Kijeja	Collected	Snack<br>Lunch	2
Vavatenina	*Oxya hyla*	Kijeja	Collected	Snack<br>Lunch	3
Farafangana	*Locusta migratoria*	Ankraoka	Collected or sold during outbreak (1000 MgA/Kapoaka)	Snack<br>Lunch	4
Antsohihy	*Brachytrupes membranaceus*	Sahobaka	Collected or sold (200 MgA/piece)	Snack<br>Lunch	4

The recipes

Ingredients

- Fresh grasshopper
- Water (optional)
- Salt (optional)
- Oil (optional)

Preparations

Dish 1: ***Whole roasted grasshopper***

1. Wash the grasshopper
2. Spit with a skewer or fork

3. Roast directly in the fire

Dish 2: ***Wrapped roasted grasshopper***

1. Remove the wings/legs/intestine/head
2. Wash the grasshopper
3. Salt
4. Wrap with *Longosy* leaves
5. Roast directly in the fire

E.g: *Paracinema tricolor* (Figure 1) in Vavatenina. The wings and the legs are removed, then the body is washed, salted, and wrapped with *Longosy*, an edible leaf. Afterwards, the wrapped grasshopper is roasted in the fire.

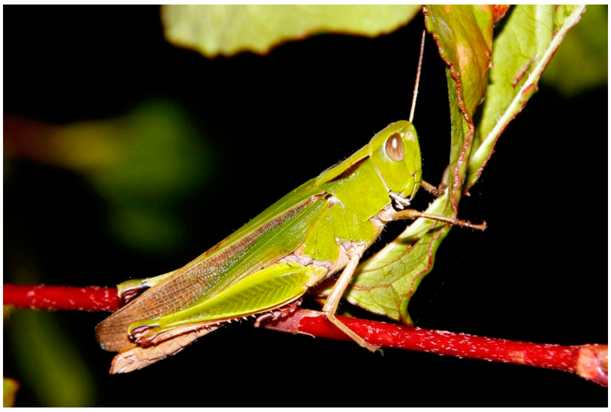

Figure 1: *Paracinema tricolor* (picture courtesy: Sylvain Hugel)

Dish 3: ***Whole fried grasshopper***

1. Wash the grasshopper
2. Put in the saucepan
3. Salt
4. Fry with/without oil

E.g: *Oxyla hyla* (Figure 2) in Vavatenina, is fried in a saucepan with salt and oil.

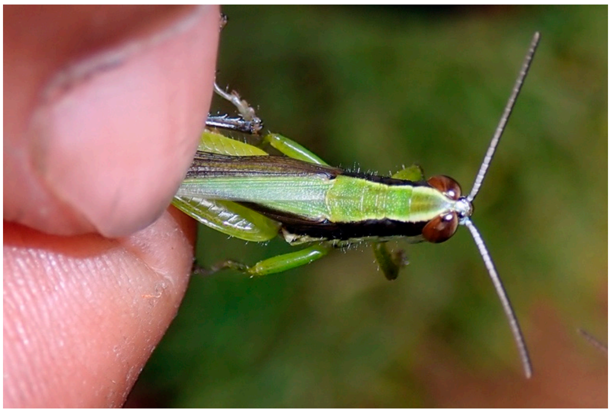

Figure 2: *Oxyla hyla* (picture courtesy: Sylvain Hugel)

Dish 4: ***Special fried grasshopper***

1. Remove the wings/legs/intestine/head
2. Wash the grasshopper
3. Put in a saucepan
4. Salt
5. Fry with/without oil

E.g 1: *Brachytrupes membranaceus* (Figure 3). The head, wings, and legs are removed before cooking in water, then fried with oil and salt.

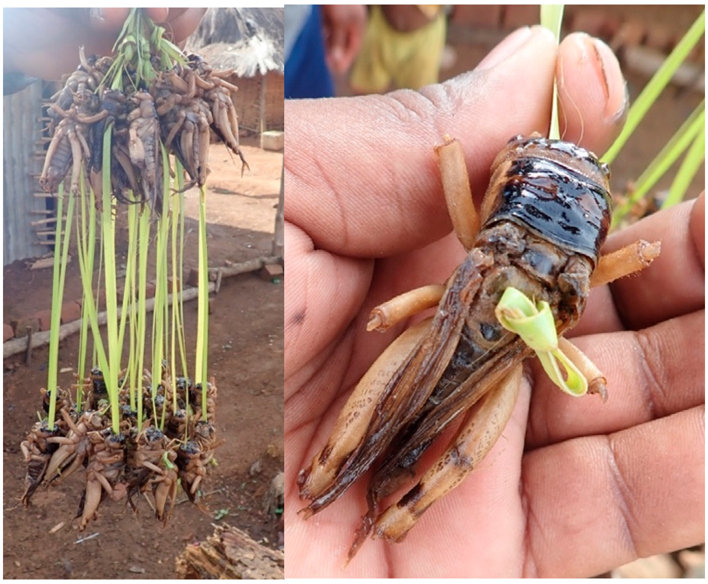

Figure 3: *Brachytrupes membranaceus* sold in the market (picture courtesy: Faneva Rajemison)

E.g 2: *Locusta migratoria* (Figure 4–8)

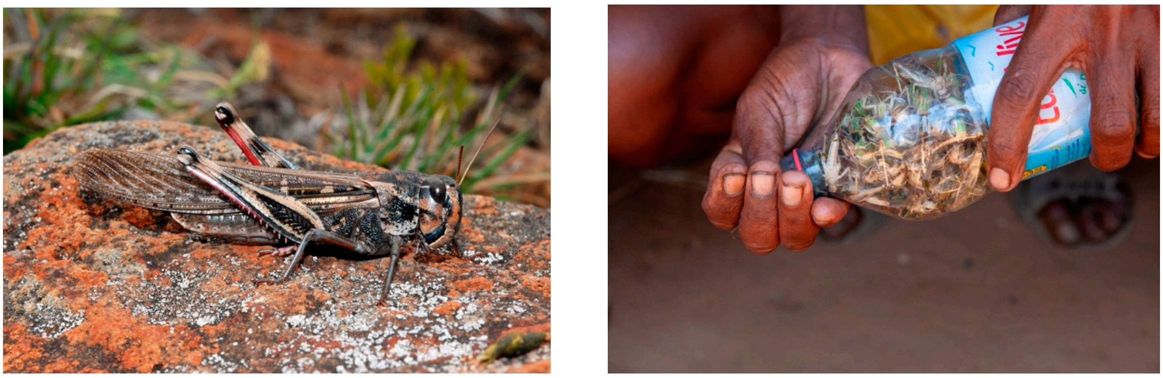

Figure 4: *Locusta migratoria* collected from the wild (picture courtesy: Sylvain Hugel – left – and Brian L. Fisher – right)

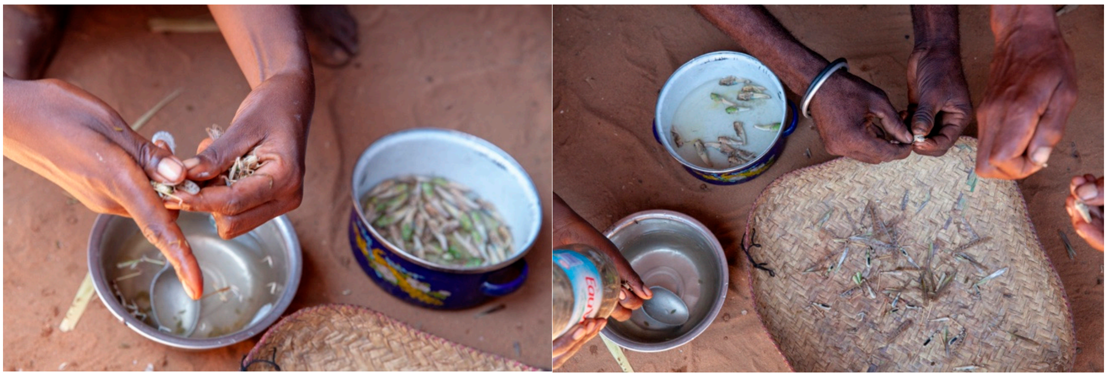

Figure 5: Removing the legs and the wings (picture courtesy: Brian L. Fisher)

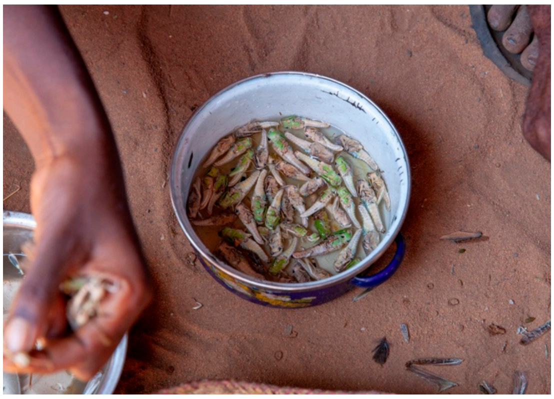

Figure 6: Washing the locust (picture courtesy: Brian L. Fisher)

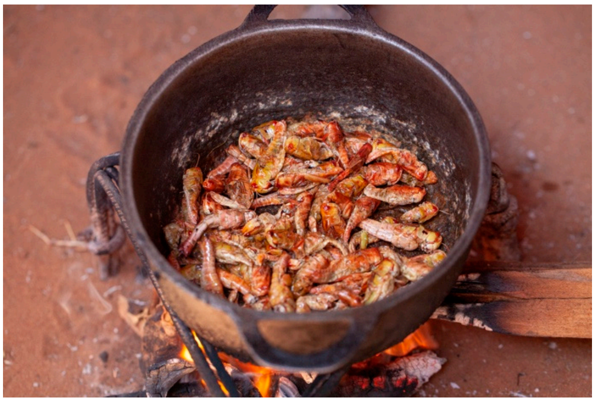

Figure 7: Frying locust in the saucepan with oil (picture courtesy: Brian L. Fisher)

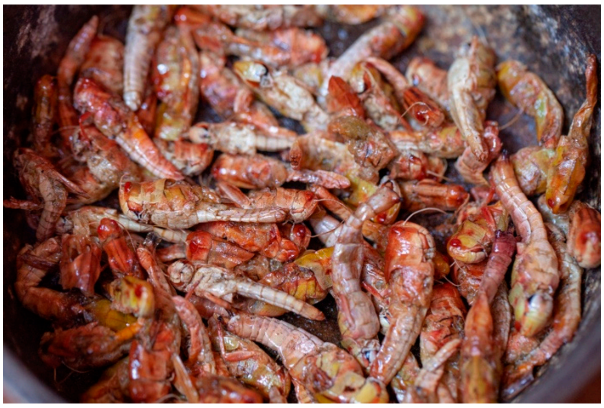

Figure 8: The locusts are ready to eat (picture courtesy: Brian L. Fisher)
